# Multi-Marker Detection of Diabetic Kidney Disease and Risk of Incident Diabetic Retinopathy in a Multi-Ethnic Asian Population

**DOI:** 10.3390/diagnostics16101492

**Published:** 2026-05-14

**Authors:** Guan Hui Yap, Barry Moses Quan Ren Koh, Miao Li Chee, Riswana Banu, Sieh Yean Kiew, Cynthia Ciwei Lim, Gavin Tan, Ching-Yu Cheng, Charumathi Sabanayagam

**Affiliations:** 1 Singapore Eye Research Institute, Singapore National Eye Centre, Singapore 169856, Singapore; yapguanhui@gmail.com (G.H.Y.); e0175068@u.duke.nus.edu (B.M.Q.R.K.); chee.miao.li@seri.com.sg (M.L.C.); riswana.banu.mohd.abdul@seri.com.sg (R.B.); kiew.sieh.yean@singhealth.com.sg (S.Y.K.); gavin.tan@singhealth.com.sg (G.T.); chingyu.cheng@duke-nus.edu.sg (C.-Y.C.); 2 Department of Renal Medicine, Singapore General Hospital, Singapore 169856, Singapore; cynthia.lim.c.w@singhealth.com.sg; 3 Ophthalmology and Visual Sciences Academic Clinical Program, Duke-NUS Medical School, Singapore 169857, Singapore

**Keywords:** serum cystatin C, chronic kidney disease, serum creatinine, albuminuria, diabetic retinopathy

## Abstract

**Background/Objectives**: Cystatin C-based and combined creatinine–cystatin C estimated glomerular filtration rate (eGFR) equations improve early chronic kidney disease (CKD) detection and prediction of adverse outcomes compared to creatinine alone. However, their role in predicting microvascular complications such as diabetic retinopathy (DR) is less clear. We examined the association between diabetic kidney disease (DKD), defined using creatinine-, cystatin C-, and combined eGFR measures, as well as albuminuria, and the risk of incident DR among Asian adults in Singapore. **Methods**: We analysed 1135 Chinese and Indian adults with diabetes aged ≥40 years from a population-based cohort study with baseline (2007–2011) and 6-year follow-up (2013–2017) data. DR was graded from retinal photographs, and incident DR was defined as new-onset at follow-up. DKD was defined as eGFR < 60 mL/min/1.73 m^2^ using eGFRcr, eGFRcys, combined eGFRcr-cys, and albuminuria (UACR ≥ 30 mg/g), assessed individually and jointly. Modified Poisson regression models adjusted for age, sex, ethnicity, diabetes duration, HbA1c, and systolic blood pressure were used to estimate relative risks (RRs). **Results**: Overall, incident DR occurred in 13.0% of participants. Among those with DKD, incidence was 18.2% (eGFRcr), 16.7% (eGFRcys), 23.7% (eGFRcr-cys), and 18.3% (albuminuria). eGFRcr-DKD (RR = 2.18, 95% CI 1.33–3.58), eGFRcys-DKD (2.38 [1.51–3.78]), and eGFRcr-cys-DKD (3.15 [1.94–5.12]) were independently associated with incident DR, whereas albuminuria alone was not. Risk increased with increasing number of markers,2.00 (1.02–3.92) by dual and 4.91 (2.50–9.65) by triple markers. **Conclusions**: DKD defined using multiple kidney markers, particularly combined creatinine–cystatin C, was strongly associated with incident DR. These findings support the use of multiple kidney function markers to improve risk stratification for developing DR.

## 1. Introduction

Diabetes mellitus affects approximately 415 million people worldwide [1], and diabetic retinopathy (DR) is the leading cause of vision loss in working-age adults [2]. DR is known to be closely related to the other microvascular complications of diabetes, in particular the presence of diabetic kidney disease (DKD) [3]. However, previous studies mostly used traditional biomarkers for DKD, such as the urinary albumin-to-creatinine ratio (urinary ACR) and estimated glomerular filtration rate (eGFR) based on serum creatinine. In recent times, cystatin C has been identified as an alternate biomarker of kidney function; unlike serum creatinine, it is not affected by muscle mass, nutritional status, age, or sex [4,5]. Studies in patients with chronic kidney disease have shown that cystatin C levels rise earlier than serum creatinine levels, suggesting that cystatin C may be more sensitive than serum creatinine as a marker of renal impairment [4,5]. Additionally, in our previous study, the combined creatinine- and cystatin C eGFR (eGFRcr-cys 2021) outperformed creatinine-based GFR equations in predicting all-cause mortality and performed comparably to cystatin C-based eGFR for predicting both all-cause mortality and incident cardiovascular disease [6].

We have previously shown in prevalence studies that elevated serum cystatin C levels were associated with DR [6], and other studies have demonstrated that elevated serum cystatin C is an independent risk factor for DR, along with diabetes duration and glycosylated haemoglobin A1c (HbA1c) levels [7]. Longitudinal evidence examining whether multi-marker definitions of DKD improve prediction of incident or progressive DR remains limited. Therefore, in this study, we examined the association between DKD (defined using creatinine- and/or cystatin C-based eGFR and albuminuria) and the risk of incident DR and progression of pre-existing DR.

## 2. Materials and Methods

### 2.1. Study Population and Design

**Figure 1:**
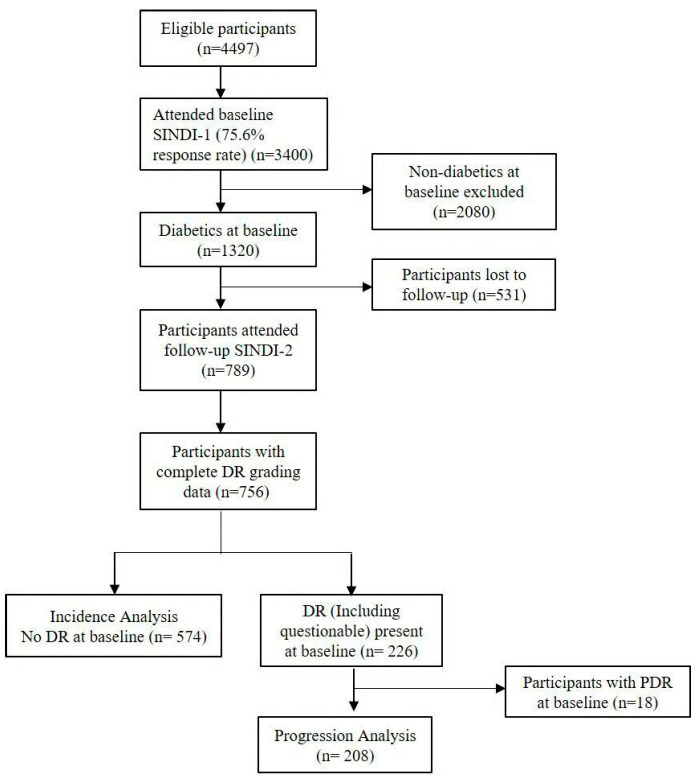
Flowchart of participants recruited from the Singapore Indian Eye Study (SINDI). Overall, 738 Indian subjects were included in DR incidence and progression. Subjects with questionable DR (n = 44) were involved in both DR incidence and progression analysis.

**Figure 2:**
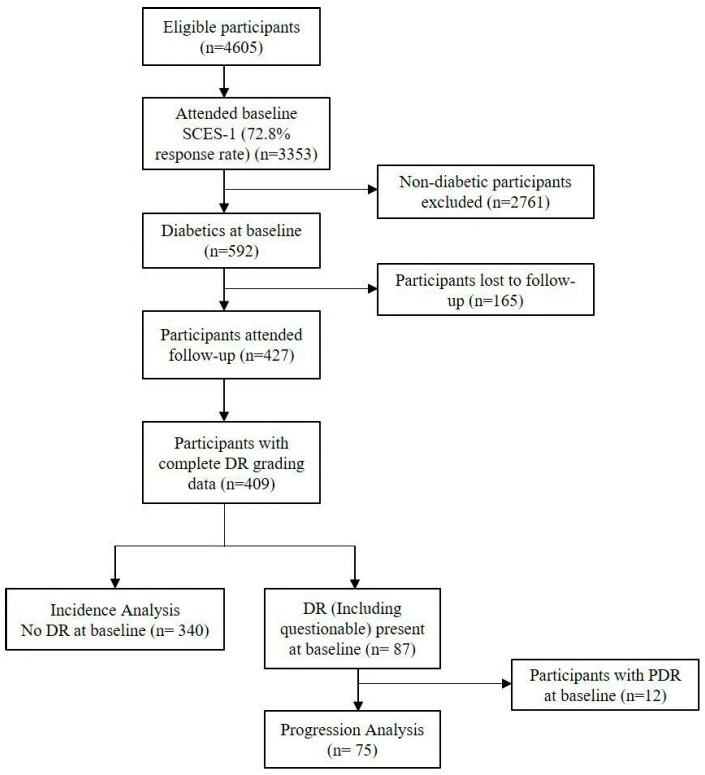
Flowchart of participants recruited from the Singapore Chinese Eye Study (SCES). Overall, 397 Chinese subjects were included in DR incidence and progression. Subjects with questionable DR (n = 18) were involved in both DR incidence and progression analysis.

This longitudinal cohort study represents a 6-year follow-up to the population-based Singapore Indian Eye Study (SINDI) and the Singapore Chinese Eye Study (SCES). At baseline (2007–2011), 6753 adults (3400 Indian individuals and 3353 Chinese individuals) aged 40–80 years residing in Singapore [8] participated in the study. Among these, 1912 had diabetes mellitus at baseline, of which 1216 attended the 6-year follow-up visit. Of these, 914 participants without DR (including questionable DR) at baseline were included for incident DR analysis, while 283 participants with pre-existing DR (including questionable DR but excluding proliferative DR (n = 30)) were included in the analysis for progression of DR. (Figure 1 and Figure 2). Of the 1912 participants with diabetes at baseline, 1216 (63.6%) attended the 6-year follow-up visit, while 696 (36.4%) were lost to follow-up. This study was supported by the Ministry of Education, Academic Research Fund Tier 1 No. T1, 1 February 2012. The study was conducted in accordance with the Declaration of Helsinki and was approved by the SingHealth Institutional Review Board (IRB Ref #-2018/2717, 2018/2570, 2018/2921, 2012/487/A, 2015/2279) on 6 January 2004, 20 November 2006, 28 June 2010, 24 July 2012, and 15 April 2015. Written informed consent was obtained from all participants.

### 2.2. Study Procedure

At both baseline and the 6-year follow-up, participants completed an interviewer-administered questionnaire to collect demographic data and underwent anthropometric measurements, including height and weight measurements. Blood pressure was measured using a digital automatic blood pressure monitor after participants were seated for at least 5 min. A comprehensive ocular examination of the anterior and posterior segments by slit lamp biomicroscopy along with fundus photography was performed using a digital nonmydriatic retinal camera (CR6-45NM, Canon, Tokyo, Japan)**.** Images were obtained according to the Early Treatment for Diabetic Retinopathy Study (ETDRS) standard fields 1 (centred on the optic disc) and 2 (centred on the fovea). At baseline, venous blood samples were collected for biochemical analyses, including serum lipid panel (total cholesterol, high-density lipoprotein cholesterol (HDL), low-density lipoprotein (LDL), triglycerides), random plasma glucose and glycosylated haemoglobin A1c (HbA1c), creatinine, and cystatin C. Spot untimed urine samples were collected for measuring urinary albumin (mg/L) and creatinine (mmol/L). Urinary creatinine in mmol/L was converted to g/L by dividing mmol/L values by 8.84, and ACR was calculated as urinary albumin divided by urinary creatinine (mg/g).

Diabetes was defined as a random blood glucose level of ≥11.1 mmol/L, glycated haemoglobin (HbA1c) ≥ 6.5%, self-reported use of antidiabetic medications, or a physician diagnosis of diabetes mellitus. The questionnaire used did not differentiate between type 1 and type 2 diabetes; type 1 diabetes was operationally defined as a diagnosis before age 30 years. Over 95% of participants reported a diagnosis at age ≥ 30 years, indicating that the cohort predominantly comprised individuals with type 2 diabetes. Based on this criterion, over 95% of participants reported a diagnosis after age 30, indicating that the overwhelming majority were classified as having type 2 diabetes. Hypertension was defined as systolic blood pressure of ≥140 mm Hg or diastolic blood pressure of ≥90 mm Hg, use of antihypertensive medication, or self-reported physician-diagnosed hypertension.

### 2.3. Assessment of Markers of DKD

CKD was defined as an eGFR of <60 mL/min/1.73 m^2^. eGFR was calculated using serum creatinine (eGFRcr) based on the CKD-EPI 2009 equation [9], serum cystatin C (eGFRcys) based on the CKD-EPI 2012 equation, and the combined creatinine–cystatin C equation (CKD-EPI 2012) [10]. Presence of albuminuria was defined as urinary ACR ≥ 30 mg/g [11,12]. Serum creatinine was measured by the Jaffe method on the Beckman DXC800 analyser calibrated to the isotope dilution mass spectrometry method using the National Institute of Standards and Technology reference material [13]. Cystatin C was measured using particle-enhanced turbidimetric assay, and urine albumin was measured using a PEG-enhanced immunoturbidimetric method on the Siemens Advia platform at a tertiary hospital laboratory (National University Health System, Singapore). DKD was defined using each of the 3 markers separately aseGFRcr-DKD, eGFRcys-DKD, and albuminuria and in combination aspresence of any single marker, any two marker, and all three markers (triple markers, eGFRcr-DKD, eGFRcys-DKD, and albuminuria).

### 2.4. Assessment of DR

Fundus photographs were graded by trained graders according to the modified Airlie House Classification system used in the ETDRS [14,15,16]. For each eye, retinopathy severity was categorised as no DR (level 10), questionable DR (level 14–15), minimal (level 20), mild non-proliferative diabetic retinopathy (NPDR; level 35), moderate NPDR (level 47), or severe NPDR (level 53) and proliferative DR (level > 60).

Based on the severity score of the worse eye, incident DR was defined as the development of any DR (ETDRS level ≥ 20) at the 6-year follow-up visit in at least one eye among participants with no DR at baseline (ETDRS level 10/10 in both eyes) [16].

Progression of DR was defined as progression of DR by an increase of ≥2 steps on the 15-step ETDRS scale over the 6-year follow-up among participants with pre-existing DR (including questionable DR, level 14/15 at baseline) and without PDR at baseline [17].

### 2.5. Statistical Analyses

All statistical analyses were performed using STATA statistical software (Version 14.1, StataCorp, College Station, TX, USA). Baseline demographic and systemic characteristics were compared between participants with and without (1) incident DR and (2) DR progression. Continuous variables were analysed using *t*-tests, and categorical variables were analysed using chi-square tests as appropriate. Associations between individual markers (eGFRcr-DKD, eGFRcys-DKD, and albuminuria) and the incidence and progression of DR were first examined using Poisson regression models with robust variance. Two models were constructed: (1) an age-, sex-, and ethnicity-adjusted model and (2) a multivariable model additionally adjusted for variables associated with incident DR or DR progression in univariable analyses (*p* < 0.05) along with established risk factors based on prior clinical knowledge. We further assessed the association between the number of renal markers and incident DR, using normal kidney function as the reference, and tested for linear trends within the same multivariable models. For incident DR, we performed subgroup analysis stratified by ethnicity. For DR progression, stratification by number of markers or by ethnicity was not conducted due to the limited number of events.

In supplementary analyses, the 2012 cystatin C-based and creatinine–cystatin C combined equations were replaced with the updated CKD-EPI 2021 creatinine–cystatin C equation, in line with recent KDIGO guidelines [18].

In addition to evaluating renal biomarkers individually, we performed a secondary analysis using eGFR estimated from the 2021 Cr-Cys equation in combination with albuminuria to evaluate whether this clinically relevant composite measure improved associations with incident DR.

The non-linearity between eGFRcr and incident diabetic retinopathy was also assessed using a modified Poisson regression model with a restricted cubic spline, adjusted for the same covariates in the above multivariable model. Lastly, we performed a comparison of baseline characteristics between diabetic subjects who followed up with those who were lost to follow-up to assess for potential attrition bias.

## 3. Results

### 3.1. Incident DR

The incidence of new-onset DR was 13.0% over the 6-year follow-up period. Baseline characteristics of participants stratified by incident DR status are presented in Table 1. Participants who developed incident DR were more likely to be younger and male, and a higher proportion were of Indian ethnicity. They also had higher baseline levels of plasma glucose, HbA1c, and diastolic blood pressure. In addition, higher creatinine-based and cystatin C-based eGFR (eGFRcr and eGFRcys) were observed among those who developed incident DR.

### 3.2. Association of DKD and Incident DR

Over a median follow-up of 6 years, incident DR occurred in 13.0% of participants. Individuals who developed DR had worse glycaemic control and higher blood pressure at baseline. Table 2 shows the association between the three markers of DKD separately and in combination with incident DR in the whole population and stratified by ethnicity. In the whole population, we found that DKD defined by all three markers had an increased risk of incident DR. Specifically, 18.2% of individuals with eGFRcr-DKD, 16.7% with eGFRcys-DKD, and 18.3% with albuminuria developed incident DR. In multivariable analysis, both eGFRcr-DKD and eGFRcys-DKD showed significant positive associations (RR = 2.18, *p* < 0.002 and 2.38, *p* < 0.001) with incident DR. Albuminuria showed a significant association (RR = 1.77, *p* = 0.001) in models adjusting for age, sex, and ethnicity, but this association was attenuated and no longer significant in the multivariable model (*p* = 0.2). DKD defined as the presence of any one of the three markers was not associated with incident DR (multivariable RR= 1.03, *p* = 0.9). However, having two or three abnormal markers was significantly associated with incident DR (multivariable RR= 2.00, *p* = 0.04 for two markers and of 4.91, *p* < 0.001 for three markers). A dose–response relationship was observed with increasing number of abnormal markers, with multivariable RR values of 2.00 for two markers (*p*-trend = 0.4) and 4.91 for three markers (*p*-trend = 0.001) in the multivariable models. In analyses stratified by ethnicity, both Indian and Chinese participants showed significantly increased risks of incident DR when all three markers were present (multivariable RR = 4.48, *p* < 0.001 for Indian individuals; 6.20, *p* = 0.005 for Chinese individuals).

Supplementary analysis using CKD-EPI 2021 equations showed similar findings (Table S2). Overall, 17.9% of individuals with eGFRcr-DKD, 16.7% with eGFRcys-DKD, and 18.3% with albuminuria developed incident DR. In multivariable analyses, both eGFRcr-DKD and eGFRcys-DKD remained significantly associated with incident DR (RR = 2.11, *p* = 0.01; 2.38, *p* < 0.001). The presence of two abnormal markers was also significantly associated with a higher risk of incident DR (RR = 2.57, *p* = 0.004). When all three markers were present, the association was stronger (RR = 4.51, *p* < 0.001). Furthermore, combining albuminuria with eGFRcr-cys derived from the 2021 creatinine–cystatin C equation further increased the risk (RR = 5.46). In analyses stratified by ethnicity, both Indian and Chinese participants continued to show significantly increased risks of incident DR when all three markers were present (Indian: RR = 4.49, *p* = 0.001; Chinese: RR = 4.88, *p* = 0.04). A similar pattern was observed when albuminuria was combined with eGFRcr-cys (Indian: RR = 5.74, *p* < 0.01; Chinese: RR = 5.37, *p* = 0.01, Table S2).

### 3.3. Non-Linear Association of eGFR (RCS Analysis)

To further explore the relationship between kidney function and incident DR, we modelled eGFRcr as a continuous variable using a restricted cubic spline with three knots in a modified Poisson regression model. Risk ratios were estimated relative to a reference value of 60 mL/min/1.73 m^2^.

As shown in Supplementary Figure S1, a non-linear association was observed between eGFRcr and incident DR. While risk remained relatively stable at lower eGFR levels, an increase in risk was observed at higher eGFR values, particularly above approximately 108 mL/min/1.73 m^2^.

### 3.4. Progression of DR

Of the 283 participants with existing DR, 74 (26.1%) had progressed by two steps or more over the 6-year follow-up period. As shown in Table S1, participants who demonstrated progression of DR were younger, had higher levels of plasma glucose, and had higher HbA1c (all *p* < 0.05).

### 3.5. Association of DKD with Progression of DR

Overall, 29.4% of subjects with eGFRcr-DKD, 35.0% with eGFRcys-DKD, and 27.3% with albuminuria developed progression of DR. Table 3 shows the associations between kidney markers and progression of DR. In the model adjusted for age, sex, and ethnicity, eGFRcr < 60 mL/min/1.73 m^2^ was not significantly associated with DR progression (RR = 1.61, *p* = 0.1); however, the association became significant after multivariable adjustment (RR = 1.85, *p* = 0.04). In contrast, eGFRcys < 60 mL/min/1.73 m^2^ was significantly associated with DR progression in the age-, sex-, and ethnicity-adjusted model (RR = 1.92, *p* = 0.02), but this association was not significant in the multivariable model (RR = 1.69, *p* = 0.05). Albuminuria was not associated with progression of DR in either the age-, sex-, and ethnicity-adjusted model (RR = 1.11, *p* = 0.6) or the multivariable model (RR = 0.86, *p* = 0.5).

Supplementary analysis using CKD-EPI 2021 equations showed a higher proportion of participants who developed DR progression (32.0% vs. 29.4%), while the proportions for eGFRcys-defined DKD and albuminuria remained similar (Table S3). However, in contrast to the findings using the CKD-EPI 2009 equation, eGFRcr < 60 defined using the CKD-EPI 2021 equation was not significantly associated with DR progression in the model adjusted for age-, sex-, and ethnicity- (RR = 1.73, *p* = 0.10), but was marginally associated after full multivariable adjustment (RR = 1.88, *p* = 0.05). Similar to the main analysis, neither eGFRcys-DKD nor albuminuria was associated with DR progression in multivariable model. eGFRcr-cys DKD was significantly associated with progression with a multivariable RR of 2.02, *p* = 0.009.

## 4. Discussion

In this multi-ethnic population-based study, we found that DKD at baseline, defined using a triple-marker panel (eGFRcr < 60 mL/min, eGFRcys < 60 mL/min, and urinary ACR ≥ 30 mg/g) was significantly associated with incident DR over the 6-year follow-up period but not with progression of DR. The strongest associations were observed when multiple markers were present, suggesting a cumulative effect of renal dysfunction on DR risk. In contrast, associations with DR progression were less consistent after multivariable adjustment. The prevalence of DKD in our study population at baseline was 8.9% based on eGFRcr < 60, 13.0% based on eGFRcys < 60, and 32.7% based on albuminuria, while 2.7% had DKD based on the triple-marker definition. These estimates are consistent with previous reports from the United States (National Health and Nutrition Examination Survey, 2014) [19].

While previous work in this area has established the use of cystatin C to define DKD [5,6] and examined its association with DR [7,8,17], these studies have largely been cross-sectional. To our knowledge, this is the first longitudinal study examining the association between cystatin C-based renal markers and incidence or progression of DR. Our results indicate that defining DKD using a triple-marker panel including cystatin C-based eGFR and albuminuria may improve identification of individuals at increased risk of developing DR and inform risk-based screening strategies.

Serum creatinine, the most commonly used marker of renal function, is influenced by non-renal factors such as age, muscle mass, sex, and nutritional status and typically rises only after substantial kidney function has been lost. In contrast, Cystatin C is less affected by these factors [5] and may detect renal impairment earlier in both acute and chronic settings [6,20]. This greater sensitivity may enable earlier identification of DKD, reflecting subclinical microvascular damage that could also manifest in the retina.

In our study, DKD as defined by dual- and triple-marker approaches was significantly associated with incident DR. These associations remained significant among Indian participants in ethnicity-stratified analyses. In contrast, limited case numbers among Chinese participants, particularly for reduced eGFRcr and eGFRcys, constrained statistical power and precluded meaningful estimation in some subgroup analyses. Both eGFRcr and eGFRcys were significantly associated with incident DR and remained significant in the Indian subgroup. These findings suggest that assessment of DKD using individual eGFRcr or eGFRcys markers or dual- or triple-marker panels may help identify individuals at higher risk of developing DR. Such risk stratification may be particularly useful in resource-limited settings, where prioritising high-risk individuals for DR screening may be more feasible than screening of all patients with diabetes.

In our study, albuminuria alone was not independently associated with incident DR after multivariable adjustment, likely due to shared pathways with established risk factors such as glycaemic control and blood pressure, which were included in the model. However, when combined with reduced eGFR, albuminuria appeared to provide complementary information. While eGFR reflects filtration capacity, albuminuria is a marker of glomerular endothelial dysfunction and increased vascular permeability [18]. The presence of abnormalities across multiple renal markers may therefore identify individuals with more advanced or diffuse microvascular damage. This likely explains the substantially higher risk observed in the triple-marker group, suggesting that albuminuria contributes to risk stratification in combination with other markers rather than acting as an independent predictor.

Progression of DR was associated with DKD defined by eGFRcr, eGFRcys, and the dual-marker panel in age-, sex-, and ethnicity-adjusted models. These findings are broadly consistent with previous studies. For example, Ajin Cho et al. [21] found that a >20% decline in eGFR (measured by creatinine clearance) was associated with increased odds of progression from NPDR to PDR (OR 2.55, *p* = 0.01), while Jeng et al. [22] found that DKD was associated with a higher risk of progression from NPDR to PDR (hazard ratio (95% CI) of 2.26 (1.68–3.03). In our study, these associations were attenuated and lost statistical significance after multivariable adjustment. This likely reflects limited statistical power due to the small number of progression events, particularly for dual- and triple-marker definitions.

In our study, higher eGFR levels were observed among participants who developed incident DR. While this may initially appear counterintuitive, we performed a restricted cubic spline analysis, which demonstrated a non-linear relationship between eGFR and DR risk, with increased risk observed at higher eGFR levels above approximately 108 mL/min/1.73 m^2^ (Figure S1). Notably, these values fall within the conventionally normal range and do not meet standard definitions of glomerular hyperfiltration (>120–130 mL/min/1.73 m^2^) [23]. This finding suggests that relatively elevated eGFR—even within the normal range—may reflect early renal or systemic vascular changes associated with increased risk of microvascular complications. However, caution is warranted in interpretation. eGFR estimates are less precise at higher levels of kidney function, and a single baseline measurement may not capture longitudinal changes. In addition, residual confounding may contribute to this association. Therefore, rather than indicating true hyperfiltration, our findings support a non-linear association between kidney function and DR risk, which warrants further investigation in studies with repeated measurements.

When the CKD-EPI 2021 equation was applied [18] instead of the 2009 version, the overall pattern of associations between DKD markers and DR outcomes remained consistent, although the strength of several associations was slightly reduced. As shown in Supplementary Tables S2 and S3, the proportion of participants classified as having eGFRcr-DKD decreased slightly from 18.2% (CKD-EPI 2009) to 17.9% (CKD-EPI 2021). Overall, the associations between eGFR-based DKD (including cystatin C-based and combined markers) and incident DR remained consistent across equations, with similar effect sizes and levels of statistical significance. The CKD-EPI 2021 equation, which removes race as a variable and incorporates both creatinine and cystatin C, has been shown to improve accuracy and equity in GFR estimation [18].

With the inclusion of the combined eGFR Cr-Cys equation, the proportion of participants classified as having eGFR-DKD increased from 18.2% (CKD-EPI 2009) and 17.9% (CKD-EPI cr 2021) to 23.7% (CKD-EPI 2021 cr-cys), rising further with the addition of albuminuria to 37.5%. This likely reflects the greater sensitivity of the combined equation in detecting early or mild reductions in kidney function. Cystatin C is less influenced by muscle mass, diet, and demographic factors compared to creatinine [5], and it may better capture subtle declines in eGFR. As a result, the combined equation may reclassify individuals with borderline renal function into the DKD category, particularly those with early microvascular damage. The further increase observed with the addition of albuminuria was expected, as albuminuria represents an independent marker of glomerular injury and may precede measurable declines in eGFR.

For DR progression, a slightly higher proportion of participants with eGFRcr-defined DKD developed progression when using the 2021 equation compared with the 2009 equation. However, the association with DR progression became only marginally significant in the multivariable model when the 2021 creatinine-only equation was used. This attenuation is likely related to limited statistical power due to the small number of progression events, rather than a meaningful change in the underlying association, as the effect estimates were largely unchanged between the two equations. In contrast, the creatinine–cystatin C combined equation remained significantly associated with DR progression. As a more precise marker of kidney dysfunction [18], the combined equation may better identify individuals with underlying microvascular dysfunction, thereby strengthening the observed association. Renal markers such as eGFR and albuminuria may fluctuate due to factors including blood pressure, medications, and intercurrent illness. In contrast, progression of DR was defined using a ≥2-step increase on the ETDRS scale, which reflects structural retinal changes that are less likely to be reversible. Therefore, our findings should be interpreted as associations between baseline kidney function and subsequent DR risk, rather than indicating parallel or causal progression of disease.

The strengths of this study include the large population-based cohort with longitudinal follow-up over six years, our objective assessment of DR using retinal photography and comprehensive evaluation of DKD using a triple-marker panel (creatinine, cystatin C, and albuminuria), and the availability of data on many potential confounders for adjustment in the analyses. Some limitations should be considered when interpreting the findings of this study. First, the number of participants with incident DR or DR progression who met the triple-marker criteria was small, which may have limited statistical power and the precision of estimates. Second, approximately 36% of participants with diabetes at baseline did not attend the 6-year follow-up, raising the possibility of attrition bias. Participants lost to follow-up may have had more severe systemic or ocular disease, including advanced DR, visual impairment, or end-stage kidney disease, which could have limited their ability to return for follow-up examinations. If so, this may have resulted in an underestimation of the true incidence of DR and attenuated the observed associations between diabetic kidney disease and DR outcomes. However, such loss to follow-up is common in long-term population-based cohort studies, and our follow-up rate is comparable to similar epidemiological studies. Future studies incorporating linkage to clinical registries or mortality data may help better account for outcomes among participants lost to follow-up. Third, Malay participants were excluded due to the unavailability of cystatin C measurements in this group. As Malays represent a high-risk population for CKD, this may limit the generalisability of our findings. Future studies including Malay participants are needed to better understand the relationship between multi-marker DKD definitions and DR outcomes across all major ethnic groups. Fourth, missing data were present for DKD, ranging from 2% for eGFR creatinine to 11% for cystatin C. Although this is not uncommon in cohort studies, missing data may introduce selection biases if individuals without DKD data differs systematically from those included in analysis. Future prospective studies with more complete DKD data capture are needed to validate our findings, as missing data may potentially result in overestimated or underestimated effect sizes (Table S4). Lastly, differential loss to follow-up may have introduced survivor bias. In our analysis, participants lost to follow-up had a less favourable baseline risk profile, including older age, higher systolic blood pressure, poorer glycaemic control, and worse kidney function. This suggests that individuals at higher risk of microvascular complications may have been underrepresented at follow-up, potentially due to morbidity and mortality. As a result, the incidence of DR and the observed associations between diabetic kidney disease and DR outcomes may have been underestimated. Therefore, our findings should be interpreted as potentially conservative estimates of the true effect.

## 5. Conclusions

In conclusion, DKD defined using a triple-marker panel was strongly associated with an increased risk of incident and progressive DR among Indian and Chinese adults with diabetes. If confirmed in future studies, incorporating cystatin C-based eGFR alongside creatinine-based eGFR and albuminuria may improve risk stratification and help identify individuals at higher risk for DR who may benefit from closer surveillance and timely intervention to prevent sight-threatening complications. Further studies are needed to validate these findings and determine the clinical utility of this approach.

## Figures and Tables

**Table 1:** Univariate comparison of baseline characteristics between participants with and without incident diabetic retinopathy status at follow-up visit.

	Incident DR, No (n = 795)	Incident DR, Yes (n = 119)	*p*-Value
Age, years	60.2 (9.3)	57.5 (9.8)	0.004
Sex, female	386 (48.6)	43 (36.1)	0.011
Ethnicity			
Indian	484 (60.9)	90 (75.6)	0.002
Chinese	311 (39.1)	29 (24.4)	
Body mass index (BMI)	26.7 (4.6)	26.1 (4.0)	0.1
Current smoking	97 (12.2)	18 (15.1)	0.4
Systolic blood pressure (BP), mm Hg	138.0 (18.1)	140.5 (17.8)	0.2
Diastolic blood pressure (BP), mm Hg	77.3 (9.5)	79.7 (8.8)	0.01
Random blood glucose, mmol/L	8.9 (3.6)	11.2 (4.4)	<0.001
Glycated haemoglobin, (HbA1c), mmol/L	7.1 (1.2)	8.3 (1.7)	<0.001
Total cholesterol, mmol/L	5.0 (1.1)	4.9 (1.0)	0.6
High density lipoprotein (HDL) cholesterol, mmol/L	1.1 (0.3)	1.0 (0.3)	0.1
Low density lipoprotein (LDL) cholesterol, mmol/L	3.0 (0.9)	3.1 (0.9)	0.7
Creatinine-based eGFR (eGFRcr), mL/min	86.6 (17.6)	90.2 (20.2)	0.04
Cystatin C-based eGFR (eGFRcys), mL/min	84.8 (20.9)	89.9 (23.3)	0.02
Urinary albumin-to-creatinine ratio (UACR), mg/g	50.3 (182.4)	57.3 (108.7)	0.7

Abbreviations: eGFR, estimated glomerular filtration rate. Data are presented as numbers and (proportions) or mean and (SD). *p*-values represent differences in characteristics based on χ2 test or *t*-test as appropriate. *p* < 0.05 denotes statistically significant results.

**Table 2:** Association between markers of DKD and incident diabetic retinopathy (DR) in the study population.

	Number at Risk, N	Number of Cases, n (%)	Age, Sex, Ethnicity Adjusted, RR (95% CI)	*p*-Value	Multivariable, RR (95% CI) *	*p*-Value
**Individual markers**						
eGFRcr ≥ 60	817	103 (12.6)	Reference		Reference	
eGFRcr < 60	77	14 (18.2)	1.87 (1.11–3.17)	0.02	2.18 (1.33–3.58)	0.002
eGFRcys ≥ 60	712	92 (12.9)	Reference		Reference	
eGFRcys < 60	102	17 (16.7)	1.73 (1.07–2.77)	0.02	2.38 (1.51–3.78)	<0.001
Albuminuria, no	597	67 (11.2)	Reference		Reference	
Albuminuria, yes	257	47 (18.3)	1.77 (1.26–2.48)	0.001	1.27 (0.89–1.83)	0.2
**Combination of markers**						
No DKD	497	58 (11.7)	Reference		Reference	
Any single marker	246	36 (14.6)	1.36 (0.93–1.99)	0.1	1.03 (0.70–1.51)	0.9
Any 2 markers	46	7 (15.2)	1.82 (0.90–3.71)	0.1	2.00 (1.02–3.92)	0.04
All 3 markers	21	8 (38.1)	5.40 (2.70–10.79)	<0.001	4.91 (2.50–9.65)	<0.001
P-trend				<0.001		0.001
**Associations stratified by ethnicity**						
**Indian**						
eGFRcr ≥ 60	517	79 (15.3)	Reference		Reference	
eGFRcr < 60	46	10 (21.7)	1.80 (0.98–3.31)	0.06	2.09 (1.17–3.72)	0.01
eGFRcys ≥ 60	457	72 (15.8)	Reference		Reference	
eGFRcys < 60	69	14 (20.3)	1.70 (1.02–2.84)	0.04	2.47 (1.51–4.03)	<0.001
Albuminuria, no	389	53 (13.6)	Reference		Reference	
Albuminuria, yes	142	34 (23.9)	1.78 (1.21–2.61)	0.003	1.23 (0.81–1.87)	0.3
**Combination of markers**						
No DKD	326	46 (14.1)	Reference		Reference	
Any single marker	162	28 (17.3)	1.33 (0.87–2.05)	0.2	0.99 (0.64–1.51)	0.9
Any 2 markers	28	7 (25.0)	2.29 (1.12–4.66)	0.02	2.57 (1.32–4.99)	0.005
All 3 markers	10	5 (50.0)	5.20 (2.24–12.04)	<0.001	4.48 (2.00–10.02)	<0.001
P-trend				<0.001		0.005
**Chinese**						
eGFRcr ≥ 60	300	24 (8.0)	Reference		Reference	
eGFRcr < 60	31	4 (12.9)	2.09 (0.73–5.98)	0.2	2.33 (0.81–6.65)	0.1
eGFRcys ≥ 60	255	20 (7.8)	Reference		Reference	
eGFRcys < 60	33	3 (9.1)	1.86 (0.55–6.24)	0.3	2.05 (0.58–7.25)	0.3
Albuminuria, no	208	14 (6.7)	Reference		Reference	
Albuminuria, yes	115	13 (11.3)	1.77 (0.86–3.63)	0.1	1.41 (0.66–2.99)	0.4
**Combination of markers**						
No DKD	171	12 (7.0)	Reference		Reference	
Any single marker	84	8 (9.5)	1.43 (0.61–3.37)	0.4	1.12 (0.46–2.73)	0.8
Any 2 markers	18	0 (0.0)	n.a.	n.a.	n.a.	n.a.
All 3 markers	11	3 (27.3)	5.62 (1.73–18.25)	0.004	6.20 (1.76–21.82)	0.005
P-trend				0.067		0.2

Abbreviations: eGFRcr, estimated glomerular filtration rate (creatinine-based calculation); eGFRcys, estimated glomerular filtration rate (cystatin C-based calculation); RR, risk ratio; urinary ACR, urinary albumin-to-creatinine ratio. Missing data: eGFRcr (n = 20; 2.2%); eGFRcys (n = 100; 10.9%); albuminuria (n = 60; 6.6%). n.a: not applicable in view of zero events. * statistically significant, *p* < 0.05. Multivariable modified Poisson regression models adjusted for age, sex, ethnicity, duration of diabetes, HbA1c, and systolic blood pressure. Bold text indicates subgroup headers (Indian/Chinese) and markers categories (individual/combination of markers).

**Table 3:** Association between markers of DKD and diabetic retinopathy (DR) progression in study population (includes questionable DR).

	Number at Risk, N	Number of Cases, n (%)	Age, Sex, Ethnicity Adjusted, RR (95% CI)	*p*-Value	Multivariable, RR (95% CI) *	*p*-Value
**Individual markers**						
eGFRcr ≥ 60	238	60 (25.2)	Reference		Reference	
eGFRcr < 60	34	10 (29.4)	1.61 (0.88–2.94)	0.1	1.85 (1.03–3.34)	0.04
eGFRcys ≥ 60	216	52 (24.1)	Reference		Reference	
eGFRcys < 60	40	14 (35.0)	1.92 (1.13–3.28)	0.02	1.69 (0.99–2.88)	0.05
Albuminuria, no	148	37 (25.0)	Reference		Reference	
Albuminuria, yes	110	30 (27.3)	1.11 (0.74–1.68)	0.6	0.86 (0.56–1.31)	0.5

Abbreviations: eGFRcr, estimated glomerular filtration rate (creatinine-based calculation); eGFRcys, estimated glomerular filtration rate (cystatin C-based calculation); RR, risk ratio. * statistically significant, *p* < 0.05. Missing data: eGFRcr (n = 11; 3.9%); eGFRcys (n = 27; 9.5%); albuminuria (n = 25; 8.8%). Multivariable modified Poisson regression models adjusted for age, sex, ethnicity, duration of diabetes, HbA1c, and systolic blood pressure. Bold text indicates markers categories (individual/combination of markers).

## Data Availability

As this study involved human participants, the data cannot be made freely available in the manuscript, the supplemental files, or a public repository due to ethical restrictions. Nevertheless, the data are available from the Singapore Eye Research Institutional Ethics Committee for researchers who meet the criteria for access to confidential data. Interested researchers can send data access requests to the Singapore Eye Research Institute using the following email address: seri@seri.com.sg.

## Supplementary Materials

The following supporting information can be downloaded at: https://www.mdpi.com/article/10.3390/diagnostics16101492/s1, Table S1: Comparison of demographic data of subjects with and without DR progression (including questionable DR) at follow-up visit; Table S2: Association between markers of DKD and incident diabetic retinopathy (DR) in the study population; Table S3: Association between markers of DKD and diabetic retinopathy (DR) progression in study population (Includes questionable DR); Table S4: Comparison of baseline characteristics between subjects who attended vs. subjects who were lost to follow-up; Figure S1: Non-linear association between eGFRcr (restricted cubic spline, three knots) and incident diabetic retinopathy.
