# Supplementary material for: Multi-Marker Detection of Diabetic Kidney Disease and Risk of Incident Diabetic Retinopathy in a Multi-Ethnic Asian Population
Source: Diagnostics (Basel). 2026 May 14;16(10):1492. doi: 10.3390/diagnostics16101492 (PMC13206007; doi:10.3390/diagnostics16101492)
Supplement: Supplementary file 1 [file diagnostics-16-01492-s001.zip › diagnostics-4266482-supplementary.pdf]

SUPPLEMENTARY MATERIALS

Table S1. Comparison of demographic data of subjects with and without DR progression (including questionable DR) at follow-up visit.

	No progression (n =209)	Progression (n = 74)	P-value
Age, years	61.1 (9.1)	58.3 (9.6)	0.03
Sex, Female	88 (42.1)	35 (47.3)	0.4
Ethnicity			
Indian	151 (72.2)	57 (77.0)	0.4
Chinese	58 (27.8)	17 (23.0)	
Body mass index (BMI)	25.8 (4.3)	26.9 (5.9)	0.09
Current smoking	23 (11.0)	8 (10.8)	> 0.99
Systolic blood pressure (BP), mm Hg	142.4 (19.6)	144.8 (21.7)	0.4
Diastolic blood pressure (BP), mm Hg	76.6 (9.6)	78.4 (11.0)	0.2
Random blood glucose, mmol/L	9.7 (4.0)	12.1 (5.5)	<0.001
Glycated haemoglobin, (HbA1c), mmol/L	7.6 (1.4)	8.8 (1.9)	<0.001
Total cholesterol, mmol/L	4.7 (1.2)	4.9 (1.3)	0.3
HDL, mmol/L	1.1 (0.3)	1.0 (0.3)	0.4
LDL, mmol/L	2.8 (0.9)	3.0 (1.1)	0.2
Creatinine-based eGFR (eGFRcr), mL/min	84.6 (17.2)	86.9 (20.9)	0.4
Cystatin C-based eGFR (eGFRcys), mL/min	84.3 (21.2)	85.4 (24.7)	0.7
Urinary albumin-to-creatinine ratio (UACR), mg/g	102.5 (417.6)	200.9 (1011.9)	0.3

Data presented are numbers and (proportions) or mean and (SD).

P value represents difference in characteristics based on χ^2 test or t-test as appropriate.

$p < 0.05$ denotes statistically significant results.

Table S2. Association between markers of DKD and incident diabetic retinopathy (DR) in the study population

	Incident DR					
	Number at risk<br>N	Number of cases<br>n (%)	Age, sex, ethnicity-adjusted, RR (95% CI)	p-value	Multivariable, RR (95% CI)*	p-value
**Individual markers**						
eGFRcr (2021) ≥60	838	107 (12.8)	Reference		Reference	
eGFRcr (2021) <60	56	10 (17.9)	1.79 (0.97 - 3.32)	0.06	2.11 (1.18 - 3.76)	0.01
eGFRcys ≥60	712	92 (12.9)	Reference		Reference	
eGFRcys <60	102	17 (16.7)	1.73 (1.07 – 2.77)	0.02	2.38 (1.51 – 3.78)	< 0.001
Albuminuria, no	597	67 (11.2)	Reference		Reference	
Albuminuria, yes	257	47 (18.3)	1.77 (1.26 – 2.48)	0.001	1.27 (0.89 – 1.83)	0.2
**Combination of markers (with 2021 equation)**						
No DKD	500	59 (11.8)	Reference		Reference	
Any single marker	255	36 (14.1)	1.30 (0.89 - 1.90)	0.2	1.00 (0.68 - 1.47)	1.0
Any 2 markers	38	8 (21.1)	2.66 (1.36 - 5.19)	0.004	2.57 (1.36 - 4.87)	0.004
All 3 markers	17	6 (35.3)	4.98 (2.29 - 10.84)	<0.001	4.51 (2.08 - 9.78)	<0.001
P-trend				<0.001		0.003

eGFRcr-cys ≥60	755	95 (12.6)	Reference		Reference	
eGFRcr-cys <60	59	14 (23.7)	2.62 (1.58 - 4.36)	<0.001	3.15 (1.94 - 5.12)	<0.001
**Combination of markers (with eGFRcr-cys (2021))**						
No DKD	540	60 (11.1)	Reference		Reference	
Albuminuria or eGFRcr-cys <60	246	40 (16.3)	1.58 (1.10 - 2.26)	0.01	1.15 (0.79 - 1.67)	0.5
eGFRcr-cys <60 and Albuminuria	24	9 (37.5)	5.81 (3.03 - 11.16)	<0.001	5.46 (2.79 - 10.68)	<0.001
**Associations stratified by ethnicity**						
Indian						
eGFRcr (2021) ≥60	529	81 (15.3)	Reference		Reference	
eGFRcr (2021) <60	34	8 (23.5)	1.95 (0.99 - 3.82)	0.052	2.14 (1.14 - 3.99)	0.02
eGFRcys ≥60	457	72 (15.8)	Reference		Reference	
eGFRcys <60	69	14 (20.3)	1.70 (1.02 – 2.84)	0.04	2.47 (1.51 – 4.03)	< 0.001
Albuminuria, no	389	53 (13.6)	Reference		Reference	
Albuminuria, yes	142	34 (23.9)	1.78 (1.21 – 2.61)	0.003	1.23 (0.81 – 1.87)	0.3
**Combination of markers (with 2021 equation)**						
No DKD	327	46 (14.1)	Reference		Reference	
Any single marker	169	29 (17.2)	1.34 (0.87 - 2.04)	0.2	1.01 (0.66 - 1.53)	1.0

Any 2 markers	22	7 (31.8)	3.03 (1.49 - 6.14)	0.002	2.94 (1.50 - 5.78)	0.002
All 3 markers	8	4 (50.0)	5.36 (2.12 - 13.58)	<0.001	4.49 (1.80 - 11.24)	0.001
P-trend				<0.001		0.007
eGFRcr-cys ≥60	490	75 (15.3)	Reference		Reference	
eGFRcr-cys <60	36	11 (30.6)	2.60 (1.48 - 4.56)	0.001	3.15 (1.86 - 5.35)	<0.001
**Combination of markers (with eGFRcr-cys (2021))**						
No DKD	361	47 (13)	Reference		Reference	
Albuminuria or eGFRcr-cys <60	154	33 (21.4)	1.71 (1.15 - 2.54)	0.008	1.21 (0.81 - 1.82)	0.4
eGFRcr-cys <60 and Albuminuria	11	6 (54.5)	6.16 (2.87 - 13.25)	<0.001	5.74 (2.56 - 12.88)	<0.001
Chinese						
eGFRcr (2021) ≥60	309	26 (8.4)	Reference		Reference	
eGFRcr (2021) <60	22	2 (9.1)	1.31 (0.31 - 5.62)	0.7	1.88 (0.40 - 8.81)	0.4
eGFRcys ≥60	255	20 (7.8)	Reference		Reference	
eGFRcys <60	33	3 (9.1)	1.86 (0.55 – 6.24)	0.3	2.05 (0.58 – 7.25)	0.3
Albuminuria, no	208	14 (6.7)	Reference		Reference	
Albuminuria, yes	115	13 (11.3)	1.77 (0.86 – 3.63)	0.1	1.41 (0.66 – 2.99)	0.4

**Combination of markers (with 2021 equation)**						
No DKD	173	13 (7.5)	Reference		Reference	
Any single marker	86	7 (8.1)	1.16 (0.48 - 2.81)	0.8	0.93 (0.37 - 2.36)	0.9
Any 2 markers	16	1 (6.3)	1.50 (0.21 - 10.96)	0.7	1.31 (0.16 - 10.54)	0.8
All 3 markers	9	2 (22.2)	4.16 (1.05 - 16.49)	0.04	4.88 (1.12 - 21.31)	0.04
P-trend				0.1		0.3
eGFRcr-cys ≥60	265	20 (7.5)	Reference		Reference	
eGFRcr-cys <60	23	3 (13)	2.76 (0.85 - 9.01)	0.09	3.28 (0.93 - 11.64)	0.065
**Combination of markers (with eGFRcr-cys (2021))**						
No DKD	179	13 (7.3)	Reference		Reference	
Albuminuria or eGFRcr-cys <60	92	7 (7.6)	1.14 (0.47 - 2.76)	0.8	0.91 (0.35 - 2.33)	0.8
eGFRcr-cys <60 and Albuminuria	13	3 (23.1)	5.13 (1.56 - 16.83)	0.007	5.37 (1.47 - 19.57)	0.01

Abbreviations: Risk ratio; eGFRcr, estimated glomerular filtration rate (creatinine-based calculation); eGFRcys, estimated glomerular filtration rate (cystatin C-based calculation); Urinary ACR, urinary albumin:creatinine ratio

*Statistically significant, $p < 0.05$.

Multivariable modified poisson regression models adjusted for age, sex, study, duration of diabetes, HbA1c, and systolic blood pressure.

Table S3. Association between markers of DKD and diabetic retinopathy (DR) progression in study population (Includes questionable DR)

	Progression of DR					
	Number at risk N	Number of cases n (%)	Age, sex, ethnicity adjusted, RR (95% CI)	p-value	Multivariable, RR (95% CI)*	p-value
**Individual markers**						
eGFRcr (2021) ≥60	247	62 (25.1)	Reference		Reference	
eGFRcr (2021) <60	25	8 (32.0)	1.73 (0.91 - 3.32)	0.1	1.88 (1.00 - 3.55)	0.05
eGFRcys ≥60	216	52 (24.1)	Reference		Reference	
eGFRcys <60	40	14 (35.0)	1.92 (1.13 - 3.28)	0.02	1.69 (0.99 - 2.88)	0.05
Albuminuria, no	148	37 (25.0)	Reference		Reference	
Albuminuria, yes	110	30 (27.3)	1.11 (0.74 - 1.68)	0.6	0.86 (0.56 - 1.31)	0.5
eGFRcr-cys ≥60	227	55 (24.2)	Reference		Reference	
eGFRcr-cys <60	29	11 (37.9)	1.98 (1.14 - 3.43)	0.02	2.02 (1.19 - 3.43)	0.009

Abbreviations: RR, Risk ratio; eGFRcr, estimated glomerular filtration rate (creatinine-based calculation); eGFRcys, estimated glomerular filtration rate (cystatin C-based calculation); Urinary ACR, urinary albumin-to-creatinine ratio;

*Statistically significant, $p < 0.05$.

Multivariable modified poisson regression models adjusted for age, sex, ethnicity, duration of diabetes, HbA1c, and systolic blood pressure.

Table S4. Comparison of baseline characteristics between subjects who attended vs subjects who were lost to follow-up

	Lost to follow-up (n = 696)	Follow-up (n = 1,216)	p-value
Age, years	63.6 (10.6)	60.1 (9.4)	<0.001
Sex, Female	325 (46.7)	571 (47.0)	0.9
Ethnicity			
Indian	531 (76.3)	789 (64.9)	<0.001
Chinese	165 (23.7)	427 (35.1)	
Body mass index (BMI)	26.3 (4.9)	26.5 (4.5)	0.2
Current smoking	93 (13.4)	149 (12.3)	0.5
Systolic blood pressure (BP), mm Hg	143.1 (21.2)	139.6 (18.8)	<0.001
Diastolic blood pressure (BP), mm Hg	76.7 (10.7)	77.3 (9.5)	0.2
Random blood glucose, mmol/L	10.2 (5.5)	9.5 (4.1)	0.003
Glycated haemoglobin, (HbA1c), mmol/L	7.6 (1.6)	7.5 (1.4)	0.02
Total cholesterol, mmol/L	4.9 (1.2)	4.9 (1.1)	0.7
High density lipoprotein (HDL) cholesterol, mmol/L	1.1 (0.4)	1.1 (0.3)	0.08
Low density lipoprotein (LDL) cholesterol, mmol/L	3.0 (1.0)	3.0 (0.9)	0.9
Creatinine-based eGFR (eGFRcr), mL/min	79.1 (25.2)	86.2 (18.5)	<0.001
Cystatin-C-based eGFR (eGFRcys), mL/min	84.3 (21.2)	85.4 (24.7)	0.7
Urinary albumin-to-creatinine ratio (UACR), mg/g	102.5 (417.6)	200.9 (1011.9)	0.3

Data presented are numbers and (proportions) or mean and (SD).

P value represents difference in characteristics based on χ^2 test or t-test as appropriate.

$p < 0.05$ denotes statistically significant results.

Figure S1. Non-linear association between eGFRcr (restricted cubic spline, three knots) and incident diabetic retinopathy.

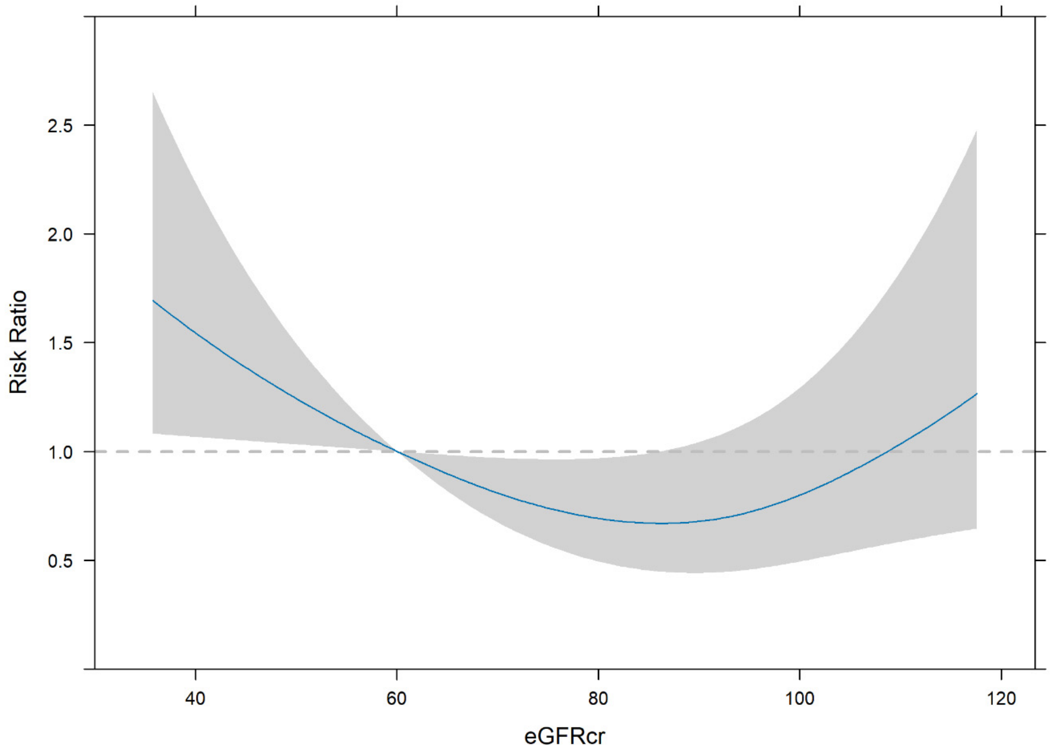

Risk ratios are plotted relative to a reference value of 60 mL/min/1.73 m². The shaded area represents 95% confidence intervals.

DR incidence Risk Ratio compared to eGFRcr = 60 mL/min/1.73 m²
